# Development and testing of locally-produced ready-to-use therapeutic and supplementary foods (RUTFs and RUSFs) in Cambodia: lessons learned

**DOI:** 10.1186/s12889-019-7445-2

**Published:** 2019-08-30

**Authors:** Bindi Borg, Seema Mihrshahi, Arnaud Laillou, Sanne Sigh, Daream Sok, Remco Peters, Chhoun Chamnan, Jacques Berger, Sophonneary Prak, Nanna Roos, Mark Griffin, Frank T. Wieringa

**Affiliations:** 1 School of Public Health, Faculty of Medicine, University of Sydney, Sydney, Australia; 2 Child Survival and Development Section, UNICEF, Phnom Penh, Cambodia; 3 Department of Nutrition, Exercise and Sports, Faculty of Science, University of Copenhagen, Copenhagen, Denmark; 4 Department of Fisheries Post-Harvest Technologies and Quality Control, Fisheries Administration, Ministry of Agriculture, Forestry and Fisheries, Phnom Penh, Cambodia; 5 Nuffield Centre for International Health and Development, Leeds Institute of Health Sciences, Faculty of Medicine and Health, University of Leeds, Leeds, UK; 6 UMR Nutripass, Institut de Recherche pour le Développement, IRD/UM/SupAgro, Montpellier, France; 7 Ministry of Health, Phnom Penh, Cambodia; 8 School of Public Health, University of Queensland, Brisbane, Australia

**Keywords:** Ready-to-use supplementary food (RUSF), Ready-to-use therapeutic food (RUTF), Lipid-based nutrient supplement (LNS), Locally-produced, Childhood malnutrition, Process, Lessons learned

## Abstract

**Background:**

Rates of childhood undernutrition are persistently high in Cambodia. Existing ready-to-use supplementary and therapeutic foods (RUSFs and RUTFs) have had limited acceptance and effectiveness. Therefore, our project developed and trialled a locally-produced, multiple micronutrient fortified lipid-based nutrient supplement (LNS) with therapeutic and supplementary versions. This ready-to-use food (RUF) is innovative in that, unlike many RUFs, it contains fish instead of milk. Development began in 2013 and the RUF was finalised in 2015. From 2015 until the present, both the RUTF and the RUSF versions were trialled for acceptability and effectiveness.

**Methods:**

This paper draws on project implementation records and semi-structured interviews to describe the partnership between the Cambodian Ministries of Health and Agriculture, Forestry and Fisheries, UNICEF, the French National Research Institute for Sustainable Development (IRD), universities, and Vissot factory. It discusses the project implementation and lessons learned from the development and trialling process, and insights into positioning nutrition on the health agenda in low and middle-income countries.

**Results:**

The lessons learned relate to the importance of project planning, management, and documentation in order to seize opportunities in the research, policy, advocacy, and programming environment while ensuring adequate day-to-day project administration and resourcing.

**Conclusions:**

We conclude that projects such as ours, that collaborate to develop and test novel, locally-produced RUTFs and RUSFs, offer an exciting opportunity to respond to both local programmatic and broader research needs.

## Background

There is a longstanding recognition that undernutrition is not only an individual problem but has ramifications for economic development in many lower and middle income countries, including Cambodia [1, 2]. This has raised the profile of undernutrition, resulting in a body of evidence and agreed frameworks for addressing the problem [3]. Despite rapid economic development in Cambodia, rates of childhood undernutrition remain unacceptably high. There were significant improvements in nutrition between Cambodia’s first and second Demographic and Health Surveys (CDHS) in 2000 and 2005. In that period, the prevalence of stunting in children under 5 years dropped from 50 to 43%, wasting decreased from 17 to 8%, and underweight dropped from 39 to 28% [4, 5]. By 2010, progress in combatting child undernutrition had stalled, with prevalences of stunting, wasting and underweight in children under 5 years at 40, 11, and 28% respectively [6]. Cambodia was not on track to meet its Millennium Development Goal targets. In 2014, almost one-third (32%) of all children under 5 years were stunted, 10% were wasted, 24% were underweight, and 2% were severely acutely malnourished, with a weight-for-height z-score (WHZ) of less than − 3 [7]. This can mostly be attributed to sub-optimal infant and young child feeding practices [8, 9], as well as infection [10], that result in inadequate energy and nutrient intakes to achieve optimal growth and micronutrient status from 6 to 23 months. Our project, a nutrition-specific intervention for treating and preventing malnutrition, grew out of that context.

Over the past two decades, various products and approaches for the prevention and treatment of childhood undernutrition have been developed and tested. Special nutritious foods can be used to prevent and treat undernutrition [11–13]. Some of these energy-dense foods require preparation e.g. fortified blended products, such as Corn-Soy Blend++ (CSB++, now called Supercereal Plus), that is cooked with water to make a porridge. Alternatively, they may be ready to eat. These include compressed bars or biscuits, such as BP-5™ or BP-100™. Increasingly, ready-to-use foods are lipid-based nutrient supplements (LNSs) which are often pastes, such as the peanut-based Plumpy’Doz™ or Plumpy’Nut®. These LNSs are proving effective, thanks to their relatively higher energy content, longer shelf life, and greater convenience [13, 14]. The WHO/UNICEF protocols for the composition of ready-to-use therapeutic foods (RUTFs) and their use in the treatment of severe acute malnutrition (SAM) have demonstrated their effectiveness [15–19]. As yet, no such standardised approach exists for the formulation of ready-to-use supplementary foods (RUSFs), or for approaches to prevention of undernutrition [15, 20].

WHO and various researchers have recommended the development of new therapeutic and supplementary foods that are affordable, acceptable and effective, and their comparison with existing products in terms of their potential for preventing growth faltering and undernutrition [13, 15, 17, 20–25]. A number of existing RUSFs and RUTFs and other supplements have been used or trialled in Cambodia, but to date, their success has been limited by low acceptability and effectiveness. Thus, the development of novel ready-to-use foods (RUFs) also responds to Cambodia’s particular programmatic need [12].

UNICEF is mandated to support the Ministry of Health (MoH) to treat SAM, and to date, that had included paying for the majority of imported therapeutic product and in-patient treatment of SAM. The long-term objective, however, was that the MoH would purchase the therapeutic product themselves. Until then, the therapeutic food used to treat SAM had been BP-100™, which had limited acceptability [26]. Plumpy’Nut® had been trialled in Cambodia in 2009 and was poorly accepted [27], as elsewhere in the region [28]. In 2013, the MoH indicated that they would be more willing and able to commit to procuring therapeutic food if a cheaper, more acceptable (thus more effective) product could be purchased locally.

UNICEF was familiar with the success of a locally-produced specialised food that had been developed in Vietnam. In 2009, the Vietnamese National Institute of Nutrition, in collaboration with UNICEF and the French National Research Institute for Sustainable Development (IRD) had developed a food called HEBI (High Energy Bar for IMAM – Integrated Management of Acute Malnutrition) [28]. HEBI contained mostly local ingredients (rice, soy, and mung beans) and imported milk powder. It was formulated to resemble “mooncake”, a delicacy eaten to celebrate the Vietnamese Mid-Autumn Festival, also known as the Children’s Festival. HEBI proved more acceptable than, and as effective as, Plumpy’Nut® and became widely and successfully used in Vietnam in IMAM programming [29, 30]. It was determined that a similar project could be undertaken in Cambodia. Since milk powder is expensive and has to be imported, it was decided that the novel product should replace milk with fish, which is inexpensive, readily available, and more adapted to local tastes.

Therefore, UNICEF solicited IRD’s assistance to develop a locally-produced RUTF. IRD had worked with the Department of Fisheries Post-Harvest Technologies and Quality Control (DFPTQ) in the Fisheries Administration of the Ministry of Agriculture, Forestry and Fisheries on previous nutrition research projects, including the development of a locally-produced complementary food [31]. In addition to their research capacity, DFPTQ could contribute its expertise with fish processing.

Around the same time, in June 2014, the United Nations World Food Program (WFP) in Cambodia phased out its distribution of CSB++ to children under 2 years and pregnant and lactating women. WFP Cambodia was experiencing budget constraints, and moreover, CSB++ had not been as acceptable or effective as expected [12]. Sprinkles micronutrient powders (MNP) had been distributed through the public health system, and although they proved effective in trial [32], in practice, coverage has been limited, and they have not been shown to contribute to improvements in linear growth [33–38]. Thus, there arose a gap in programming for the prevention of undernutrition, which is traditionally WFP’s mandate. Recognising an opportunity for creating a supplementary version of the RUF to prevent undernutrition, UNICEF also engaged WFP. In 2014, a letter of agreement was signed between UNICEF, MoH, WFP, IRD, and DFPTQ to develop products for prevention and treatment of undernutrition. The aim was to create a ready-to-use food (RUF) in RUTF and RUSF versions, that would prove more acceptable, effective, and cheaper than the existing products.

## Methods

### Aim

This paper aims to share the lessons learned and challenges faced in developing and trialling locally-produced RUSFs and RUTFs in a low to middle-income country, Cambodia, where unacceptable rates of child undernutrition persist, despite robust economic growth. By describing the partners involved, the development and trialling process, and the opportunities for positioning nutrition on the health agenda, we hope that this paper will prove useful to others engaging in a similar process of local RUSF and RUTF development.

### Design

**Fig. 1:**
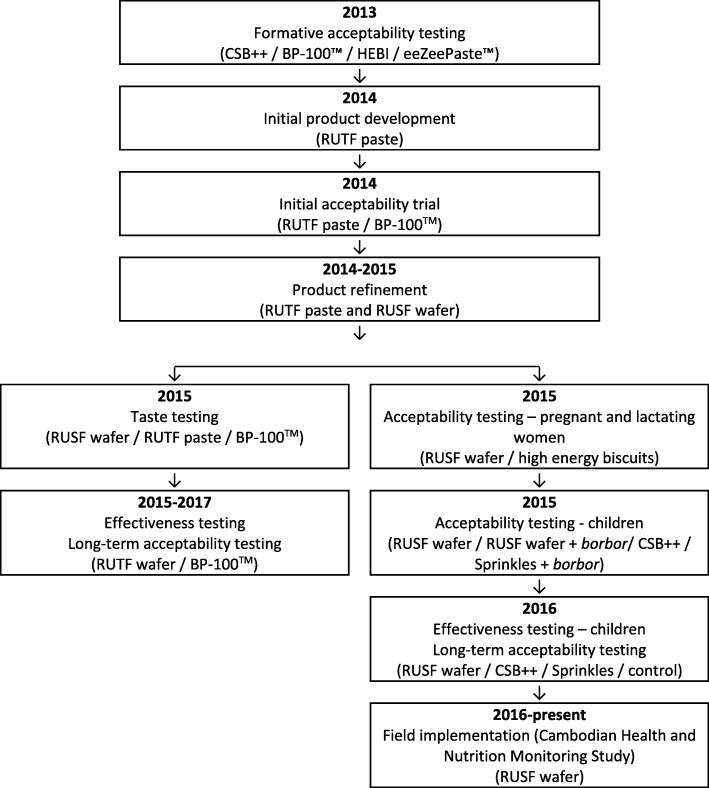
Project implementation. * Borbor: white rice porridge, the traditional Cambodian weaning food

This paper draws on project implementation records and semi-structured interviews. The project has been implemented in stages over 5 years, and is ongoing, as shown in Fig. 1. All of the trials in the project were carried out in Phnom Penh. Details on each of the trials are included under the relevant sub-headings. The trials were registered at ClinicalTrials. Gov (LNS-CAMBINFANTS, NCT02257437; LNS-CAMB-INFANTS-EFF, NCT02257762; FLNS_SAM, NCT02907424).

### Formative acceptability testing

In July 2013, IRD carried out a taste trial of CSB++, BP-100™, HEBI, and eeZeePaste™ (a peanut-based RUTF from GC Rieber Compact). Both HEBI and eeZeePaste™ proved far more acceptable than CSB++ and BP-100™ in terms of organoleptic qualities, which confirmed that the development of a locally-produced RUF adapted to the tastes of Cambodian children was warranted.

### Initial product development

In 2014, the first version of the RUF was developed [29]. Rice, small freshwater fish, soy, and mung beans - all important elements of the current Cambodian diet - were considered optimal ingredients, along with oil, sugar, and multiple micronutrients. The product was made by a local, quality-certified food factory, Vissot. Pre-tasting was conducted with Cambodian project and factory staff.

### Initial acceptability trial

In June 2014, an acceptability trial was held in a Phnom Penh preschool with 61 children aged 2 to 7 years. The trial was a 2 × 2 non-randomised, nonblinded crossover design. Children ate the novel RUF and BP-100™ for 2 weeks each. Neither the RUF nor BP-100™ were very well accepted, in terms of the amount consumed. In organoleptic scoring (of sensory qualities, e.g. taste and smell), BP-100™ scored slightly higher. Therefore, the RUF was modified to reduce the fishy taste and smell. Details on the initial product development and acceptability trial have been reported elsewhere [29].

### Product refinement

From late 2014 to early 2015, the product went through various refinements. Coconut powder was added to mask the fishy taste and smell. The form of the RUF, originally a paste, also changed. Snack consumption is ubiquitous in Cambodia, even amongst young children [12, 39, 40]. In an attempt to create a form that would be more acceptable [28], we took a well-known Cambodian snack, a wafer, and piped in the RUF paste. The final product was a wafer that is a hollow cylinder between 8.5-9 cm long with an internal diameter of 0.5 cm, filled with RUF paste. RUTF and RUSF versions were created, the main differences being the micronutrient premixes, and the oil and fibre contents. The RUFs were tested regularly for microbiological safety.

### RUSF trials

The RUSF trials included acceptability testing with children and with pregnant and lactating women in mid-2015. Effectiveness among children was tested from February to October 2016. The trials were organised with the collaboration of the staff and health volunteers of the Mekong Health District in peri-urban Phnom Penh.

#### RUSF acceptability trial – pregnant and lactating women

A non-blinded crossover study was conducted with 98 pregnant and lactating women, comparing the RUSF snack to high energy biscuits (provided by UNICEF). The women ate each food at home for 3 days, then responded to organoleptic testing. Both foods were considered highly acceptable. A planned effectiveness trial with pregnant and lactating women did not proceed, due to limited funding.

#### RUSF acceptability trial – children

A two-week, non-blinded, randomised 4 × 4 crossover trial was conducted, with 95 children aged 9–23 months. It compared the acceptability of the novel RUSF, presented as the filled wafer snack or the snack mixed into *borbor* (white rice porridge), compared to CSB++ and MNP mixed with *borbor*. Children at 4 sites ate the 4 foods for 3 consecutive days over 12 days. Although children consumed more of the MNP-*borbor*, the RUSF as a snack or mixed with *borbor* provided two to three times more kilocalories. Caregivers reported that their children had the highest preference for MNP, but that they also liked the RUSF snack. Most importantly, caregivers ranked the RUSF snack highest, and focus group discussions confirmed this. Therefore, the research team felt confident to proceed to a six-month trial to test the RUSF’s effectiveness. Details on the children’s acceptability trial are described elsewhere [41, 42].

#### RUSF effectiveness trial – children

A six-month prospective, cluster randomised, non-blinded controlled trial with a 1:1 allocation ratio was conducted with 485 healthy, non-severely acutely malnourished children aged 6 to 11 months. The aim was to establish the novel RUSF’s superiority to CSB++, MNP, and a control group. Twenty-eight sites were randomly allocated to one of the four arms. Data collection and food distribution were conducted monthly until endline. The main outcome was anthropometric status, and secondary outcomes were children’s body composition, biochemical status, and cognitive development. In addition, long-term acceptability was assessed. The RUSF was not as effective as expected. All groups continued to experience growth faltering, although the RUSF group faltered at a lower rate. Details on the effectiveness trial are described elsewhere [43], and results are forthcoming [44].

#### Cambodian Health and Nutrition Monitoring Study

The RUSF is being utilised in the Cambodian Health and Nutrition Monitoring Study. Pregnant women with a mid-upper arm circumference (MUAC) < 23 cm are deemed malnourished and provided with RUSF. While the study does not aim to trial the RUSF, it may provide additional information on the implementation, acceptability, and effectiveness of the RUSF in a programmatic setting. Results have yet to be analysed and reported.

### RUTF trials

The RUTF trial from 2015 to 2017 included taste testing to finalise the RUTF, followed by effectiveness and long-term acceptability testing with children presenting to the National Paediatric Hospital in Phnom Penh. The trial was conducted with the cooperation of the hospital staff.

#### RUTF taste testing and long-term acceptability testing

In October 2015, 52 children aged 6 months to 17 years and their caregivers participated in a taste test. These children were visiting the outpatient department for various reasons and were not necessarily malnourished. The crossover design compared BP-100™ with the RUTF paste, and the RUSF wafer. The RUTF paste was considerably less acceptable, while BP-100™ and the RUSF wafer were equally acceptable. As a result, the paste form of the RUF was abandoned, and the RUTF was finalised as a filled wafer, like the RUSF.

In the subsequent effectiveness trial (described below), long-term acceptability was assessed with severely acutely malnourished children. Both products were highly acceptable, with BP-100™ slightly more so. Acceptability of the RUTF increased over the treatment period, while acceptability of BP-100™ varied. More details on the RUTF acceptability testing are reported elsewhere [45].

#### RUTF effectiveness trial

Effectiveness was tested in a single-blinded, randomised control trial conducted from September 2015 and January 2017. A total of 121 children with uncomplicated SAM aged 6 months to 5 years were randomised to receive either the novel RUTF or BP-100™ for home consumption for a period of 8 weeks. Anthropometric measures were assessed at baseline and fortnightly until endline at the eighth week. No statistically significant differences between the two products were found for changes in anthropometric status. This suggests that the locally-produced fish-based RUTF performed as well as BP-100™ and is a potential alternative to the latter for SAM treatment in Cambodia. Details on the RUTF effectiveness trial are reported elsewhere [46].

## Results

This section describes the lessons learned from implementation of the locally-produced Cambodian RUF project. The project has provided useful insights into the opportunities and challenges of getting nutrition into the broader health and development platform in low and middle-income countries. These opportunities and challenges arise before, during and after project implementation, and emphasise the importance of having a broad overview of the project from the outset. Even before the project begins, there needs to be a deep understanding of the facilitators and obstacles in the research and policy environment, and of the experience of similar projects elsewhere. At every stage, it is vital that opportunities for uptake, advocacy, and for influencing policy or process are recognised and seized. This requires communication on multiple levels. Stakeholders must be identified, and their roles and responsibilities outlined clearly, while maintaining the balance between their respective objectives. Throughout, consistent project planning, management, resourcing, and documentation are essential.

### Research and policy environment

The global and national nutrition research, policy, and programming environment around 2010–14 gave impetus to this project. The 2010 and 2014 CDHSs [6, 7] had shown that malnutrition rates in Cambodia were not improving. WHO’s 2013 SAM guidelines had emphasised the need for research in Asia on the effectiveness of RUTFs using different ingredients, compared to existing therapeutic foods [17]. The Cambodian Fast Track Road Map for Improving Nutrition 2014–2020 acknowledged that SAM treatment needed to be expanded and accelerated and committed to developing and testing “new innovative nutrition-specific interventions, which are tailored specifically to the Cambodian context …. to improve the current strategies for the treatment and the prevention of severe malnutrition” [47]. In mid-2014, Cambodia joined the Scaling Up Nutrition (SUN) movement, thus declaring its commitment to reducing child undernutrition on the global stage. The project took advantage of this momentum.

In its turn, the project has influenced Cambodian nutrition policy by encouraging the MoH to focus on treating SAM and enabling them to do so with the novel RUTF. The existence of a locally-produced RUTF persuaded the MoH to agree to put therapeutic foods on the essential medicines list of 2017. Cambodia’s new guidelines for management of acute malnutrition (comprised of the inpatient, outpatient, and community handbooks) state that any available therapeutic product, including the locally-produced RUTF, can be used for SAM treatment, and for the management of moderate acute malnutrition [48].

On a broader level, the existence of just one RUF could rationalise integrated management of acute malnutrition. At community level, early detection could lead not only to referral of SAM children, it could also result in moderately acutely malnourished children receiving the RUSF or a low dose of the RUTF. The RUF could be either provided freely through nongovernmental organisation (NGO) programs or sold at the market. A middle model, which caregivers favour, would be for community health volunteers to sell RUF [44]. This kind of public/private production and distribution model should be explored further. Any models of distribution to non-severely acutely malnourished children must avoid inadvertently increasing the risk of overweight and obesity [49].

The project has also received attention from elsewhere in the region, specifically, Laos, Indonesia and Papua New Guinea, countries which are exploring options for developing and using their own locally-produced RUFs.

### Strategy and advocacy

The RUFs were brought to the attention of high-level Cambodian policy and decision makers in the Fill the Nutrient Gap process and report, convened by WFP [50]. By bringing together multiple ministries under the direction of the inter-ministerial Council for Agricultural and Rural  Development (CARD), the process has helped to place nutrition more firmly onto the broader government agenda. This may also facilitate nutrition-sensitive programming across sectors.

That said, at the outset, there was no clear strategy and advocacy plan for the project. Ad hoc opportunities through UNICEF events, conferences, and media were taken as they arose. Ideally, opportunities and strategies for advocacy would be identified in the planning phases of the project.

### Project communication

The project needs to be communicated to the broader group of stakeholders, especially when actors have different backgrounds, goals, and roles. Too often, research is communicated in conferences that may not be attended by a wide range of actors. The National Nutrition Program Working Group provided a forum for project communication. Events such as project launch meetings that bring together a range of actors can also facilitate communication between multiple actors and across multiple levels. An important part of effective communication, especially in hierarchical societies, entails negotiating cultural differences and protocol. On multiple levels, project communication is essential and needs to be an explicit part of the project plan.

### Stakeholders

The original group of core stakeholders comprised of UNICEF, MoH, WFP, IRD and DFPTQ provided a complementary set of skills, experience, and opportunities for developing, promoting, or utilising the RUFs. The various partners also had a history of collaboration. With respect to undernutrition, the primary mandate of UNICEF and the MoH is treating SAM, while WFP’s mandate is preventing undernutrition, including by providing supplementary food. IRD and DFPTQ provided the research skills and experience to implement the project.

It is important to be explicit about the needs and pressures on all actors, about what prerogatives are privileged or steer the project, as well as how those priorities are reconciled, and how communication will be ensured and conducted. However, the letter of agreement between the stakeholders was very general and did not outline roles or responsibilities, including resourcing. In 2015, when WFP’s funding for Cambodia decreased, and with it, the likelihood that WFP would provide supplementary food for the prevention of undernutrition in the foreseeable future, they withdrew from the project. This was a significant loss, given WFP’s expertise in the development of specialised foods. A letter of agreement that clearly outlined the roles of each stakeholder in greater detail might have assisted in the selection of the stakeholder group. Moreover, a more binding agreement might have avoided the resourcing and planning issues that impeded the project’s early progress.

### Research versus policy and program implementation

There can be tension between research and policy or program goals and timelines [51], particularly when there is a large a variety of actors (researchers and research students, national and international institutions as well as NGOs, multiple ministries and their staff and volunteers). Researchers may not appreciate the policy and implementation demands that program people face, while the latter may expect research to deliver results too quickly or definitively. On a broader level, the research that is needed to satisfy program requirements may not be the research that is considered necessary in the academic community. This project did connect universities and research agencies to UN and government agencies, but perhaps could have negotiated the complex space between research and programming more effectively by explicitly acknowledging the stakeholders’ various objectives and timelines.

### Programming

The RUTF can now be used by hospitals and health centres that provide SAM treatment, as well as by NGOs that support community-based treatment. Vissot (a certified Cambodian food manufacturer that complies with the relevant Cambodian food safety and labelling standards) is also planning to make RUF available for sale to the public.

Currently, the RUTF is being piloted on a small scale by an NGO doing community-based SAM treatment, but it is not yet being used in the health system. A major difference between HEBI and the Cambodian RUF project is that the Vietnamese government were driving the development, production, and utilisation of HEBI. Once HEBI was demonstrated to be acceptable and effective, the Vietnamese government phased out BP-100™ and began using HEBI. Thus, a green light for HEBI uptake was built into the Vietnamese process. On the other hand, in Cambodia, the government was not driving the process. Private sector production will depend on government commitment to purchase. Therefore, a green light or trigger for agreeing to procure the RUTF for use in the hospitals and health centres should have been identified and agreed upon at the early stages, either in the stakeholder letter of agreement or a project planning document.

All new business ventures face the chicken and egg dilemma – without consumers, producers find it difficult to invest, and without a product, consumers find it difficult to commit to purchasing. This project was no exception – while there was a great deal of interest in the product, including from NGOs who could use it, until the product was finalised and tested (at least for acceptability), there was no way of knowing what the demand would be, nor of knowing Vissot’s capacity to meet demand. Similarly, although there is a target price which aims to make the RUFs’ cost competitive with alternative supplementary foods, that can only be confirmed once the factory is producing at scale. Without a guaranteed demand, it was impossible to invest in the machinery and staff that would have helped the project progress in a timelier fashion. Again, a green light and procurement commitment in the stakeholder agreement may have helped to mitigate this problem.

Lessons learned are that formative research, which is seldom well-resourced, is vital. In the case of the RUF project, this would have involved project mapping which included cost analyses, and a survey or estimate of demand from NGOs as well as MoH. The National Nutrition Program Working Group comprised of government, UN agencies, researchers and NGOs working in nutrition in Cambodia, and convened by the MoH’s National Nutrition Program, could have been drawn upon to facilitate this.

### Project management

A project such as this, spanning several years, and engaging a variety of institutional stakeholders and individual actors, requires meticulous attention to daily and long-term management. It needs to continuously review the project’s clarity of purpose and roles, expected outcomes, financial and human resources, and duration. Particularly in an environment of indeterminate and multiple potential sources of funding, organisational support and staffing, the project’s plan, budget, and timing need to be defined at the outset, in order to manage expectations of all the actors. This requires an identified project manager, or if project management roles are shared, a clear division of responsibilities.

This long, multi-agency, multi-staff project also experienced challenges in project documentation, partly due to staff turnover and informal decision making. An identified project manager would be responsible for collaborating with all stakeholders to ensure thorough project documentation, including an initial project plan and regular reporting. Project documents need to outline activities, a timeline, and resources in detail. They also need to describe the research and policy context in which the project was conceived and opportunities for influencing policy, and to formulate an advocacy strategy. Decisions taken, and options excluded must be recorded.

### Project resourcing

The project team had an admirably “can-do” attitude, which yielded a high ratio of benefits for resource inputs (at least in terms of funding). The use of doctoral students (who undertook the research as part of their PhDs) reduced costs, and the embedding of the project in a government department allowed the achievement of results that went beyond what may have been achieved if roles had been too sharply defined. Conversely, the “pitch in” approach left gaps in terms of responsibilities for tasks. Similarly, a dependence on ad hoc funding that was not clearly dedicated in advance - while allowing the project to happen at all - meant that some parts (such as the effectiveness trial with pregnant and lactating women) had to be abandoned when the expected funding did not materialise.

It is essential to consider human resources and to acknowledge strengths and gaps in expertise and competence. Again, it is vital to have a defined project manager who can be responsible for tying the threads together – for calling meetings, documenting decisions, and flagging resource gaps. A project manager need not be the most senior person. Indeed, the skills of senior people and experts are too often wasted by expecting them to also do project management. Such senior people are best used as a steering committee. One of their tasks is to identify the responsibilities of the project manager, and to ensure that the manager and team members are collaborating effectively. In this way, the willingness of team members can be optimised, while ensuring that adequate documentation and project administration happen.

## Discussion

This project has responded both to a programmatic need articulated by the Cambodian MoH and to identified gaps in the current understanding of RUFs for the prevention and treatment of undernutrition. Engaging numerous actors over multiple years, it experienced challenges and successes. Most importantly, it seized an opportunity created by a combination of new research and policy and drew on similar experiences in neighbouring Vietnam. In turn, it encouraged greater commitment to sound nutrition programming and policy. Specifically, it contributed to improved guidelines for SAM treatment and created new options for nutrition programming.

The challenges could have been mitigated to a great extent by improved project planning, management, and documentation. Stakeholder agreements would have benefited from being more detailed and binding, which would have contributed to stakeholder collaboration. More rigorous project planning could have anticipated and perhaps resolved some of the dilemmas around demand, capacity, and cost of the RUFs. It would also have clearly articulated the desired policy and program outcomes, resolved tensions between research and programming, and included green lights for ensuring that the RUTF was taken up in hospitals and health clinics treating SAM. More rigorous project planning would have identified specific policy, advocacy and communication goals and opportunities in advance, rather than in an opportunistic and ad hoc fashion, thus maximising the likelihood of exploiting opportunities. At a more quotidian level, improved project management – and specifically, a designated project manager - would have mitigated some of the administrative and resourcing challenges and enabled the project to unfold more smoothly. Improved documentation would have made it easier to learn and share lessons both within the project and outside it.

## Conclusion

This collaborative project developed and tested novel, locally-produced RUTFs and RUSFs. Projects like this one can be rich and exciting in their contributions to both literature and programming. They offer fruitful opportunities for learning and exchange between research, policy, and program actors, which often go untapped. Future similar projects should focus on project planning, management and documentation that addresses both strategic (policy and advocacy) and administrative levels.

## Data Availability

Data will be made available after the publication of major outputs, upon request to the corresponding author.

## Abbreviations

CARD: Council for Agricultural and Rural Development (Cambodia)
CDHS: Cambodian Demographic and Health Survey
CSB++: Corn-Soy Blend++, now called SuperCereal Plus
DFPTQ: Department of Fisheries Post-Harvest Technologies and Quality Control (in the Fisheries Administration of the Cambodian Ministry of Agriculture, Forestry and Fisheries)
HEBI: High Energy Bar for IMAM
IMAM: Integrated Management of Acute Malnutrition
IRD: French National Research Institute for Sustainable Development
LNS: Lipid-based nutrient supplement
MoH: Ministry of Health (Cambodia)
MUAC: Mid-upper arm circumference
NGO: Nongovernmental organisation
RUF: Ready-to-use food
RUSF: Ready-to-use supplementary food
RUTF: Ready-to-use therapeutic food
SAM: Severe acute malnutrition
SUN: Scaling Up Nutrition
UN: United Nations
WFP: United Nations World Food Program
WHO: World Health Organisation
